# Optimized Design of Distributed Quasi-Cyclic LDPC Coded Spatial Modulation

**DOI:** 10.3390/s23073626

**Published:** 2023-03-30

**Authors:** Chunli Zhao, Fengfan Yang, Daniel Kariuki Waweru, Chen Chen, Hongjun Xu

**Affiliations:** 1 College of Electronic and Information Engineering, Nanjing University of Aeronautics and Astronautics, Nanjing 210016, China; 2 School of Engineering, University of KwaZulu-Natal, King George V Avenue, Durban 4041, South Africa

**Keywords:** quasi-cyclic low-density parity-check codes, spatial modulation, coded cooperation

## Abstract

We propose a distributed quasi-cyclic low-density parity-check (QC-LDPC) coded spatial modulation (D-QC-LDPCC-SM) scheme with source, relay and destination nodes. At the source and relay, two distinct QC-LDPC codes are used. The relay chooses partial source information bits for further encoding, and a distributed code corresponding to each selection is generated at the destination. To construct the best code, the optimal information bit selection algorithm by exhaustive search in the relay is proposed. However, the exhaustive-based search algorithm has large complexity for QC-LDPC codes with long block length. Then, we develop another low-complexity information bit selection algorithm by partial search. Moreover, the iterative decoding algorithm based on the three-layer Tanner graph is proposed at the destination to carry out joint decoding for the received signal. The recently developed polar-coded cooperative SM (PCC-SM) scheme does not adopt a better encoding method at the relay, which motivates us to compare it with the proposed D-QC-LDPCC-SM scheme. Simulations exhibit that the proposed exhaustive-based and partial-based search algorithms outperform the random selection approach by 1 and 1.2 dB, respectively. Because the proposed D-QC-LDPCC-SM system uses the optimized algorithm to select the information bits for further encoding, it outperforms the PCC-SM scheme by 3.1 dB.

## 1. Introduction

Multiple-input multiple-output (MIMO) is a technique that effectively improves system reliability because the signals are transmitted and received through multiple antennas [1]. A well-known MIMO technique is called vertical Bell Labs layered space–time (V-BLAST) [2]. Multiple antennas in V-BLAST are required to simultaneously transmit data at the same frequency, which makes the receiver suffer from high inter-channel interference. Further, performing the signal transmission needs inter-channel synchronization (IAS). Luckily, an emerging developed spatial modulation (SM) [3] can solve the above problems, which is due to the reason that SM only activates one antenna to transmit the data signals in each transmission slot. Additionally, in SM, the activated antenna index carries information to make the spectral efficiency enhanced. The transmission and detection process of SM has been discussed in many studies, such as [4,5].

Another technology that can effectively enhance the system reliability is cooperative diversity having source, relay and destination [6]. In cooperative communications, the relay overhears the source messages and utilizes cooperative techniques such as decode-and-forward [7] to relay the information. Compared with direct transmission, cooperative communications are able to obtain lower error probabilities, as presented in [8]. In [9,10], it was reported that the cooperative communication system based on two users can enhance the achievable rate of two users. Due to the theoretical achievements of cooperative communications, they have attracted substantial attention. A very important discovery is coded cooperation [11] (i.e., distributed channel coding) as the combination of cooperative diversity and channel codes, where channel codes are key error control technologies used to ensure reliable communication transmission. The famous LDPC code [12] can approach the Shannon limit, which raises new enthusiasm about the research of LDPC coded cooperation. In [13], the authors presented the LDPC coded cooperative system for half-duplex communications. In [14], the protograph LDPC coded cooperation for multi-relay networks was studied. In [15], the authors applied the LDPC code in the network coded cooperation and designed the network LDPC coded cooperative system with multiple users and one relay. In addition, the studies [16,17] carried out the error performance analysis for the LDPC coded cooperative scheme with orthogonal frequency-division multiplexing.

For LDPC codes, the encoding method and decoding performance are related to the parity-check matrix, so constructing the parity-check matrix is the key to the design of LDPC codes. Usually, the parity-check matrix can be constructed according to the random-like and structured methods, so LDPC codes are generally partitioned into random-like and structured LDPC codes. A special structured LDPC code is quasi-cyclic LDPC (QC-LDPC) code, where the parity-check matrix is composed of multiple square matrices (circularly-shifted identity matrix or zero matrix) with an identical size, thus reducing the computational complexity of the encoder and decoder [18]. In addition, QC-LDPC codes possess the advantages of simple encoder implementation, rapid decoding convergence and low bit error rate (BER) and have been widely used in various communication and storage schemes. Therefore, the Third-Generation Partnership Project (3GPP) has recommended QC-LDPC codes as a coding scheme for the 5G enhanced mobile broadband (eMBB) data channel [19]. In addition to the quasi-cyclic structure, the LDPC code adopted by 5G communications also adopts the so-called Raptor-like structure [20], and its parity-check matrix can be gradually expanded to one with a low code rate through a core matrix of a high code rate. In this way, 5G LDPC codes can flexibly support diverse code rates, which well adapts various rate matching and information lengths. 

Due to the diverse benefits of 5G LDPC codes, studying the distributed QC-LDPC coding (D-QC-LDPCC) scheme is very significant, where the source and relay share each other’s antennas to provide a virtual MIMO in the destination, and we can reap the benefits of MIMO technology. If each node uses multiple antennas in the D-QC-LDPCC scheme, higher link reliability is achieved. Thus, studying the D-QC-LDPCC scheme using MIMO has significant research value. Moreover, the rapid development of wireless communications increases the demand for reliable and effective communication services. Thus, effectively using the spectrum to ensure high-reliability transmission is an urgent problem to be solved. Based on this, we integrate SM into the D-QC-LDPCC system to construct the distributed QC-LDPC coded SM (D-QC-LDPCC-SM) scheme. In addition, the proper encoding approach in the relay and excellent decoding strategy at the destination are very important for improving the performance of the distributed coding scheme. Thus, in the proposed system, we effectively select partial source messages for further encoding in the relay and adopt the joint decoding method to decode the received signal at the destination. The following points show the key contributions:

- The D-QC-LDPCC-SM scheme is proposed, in which two different QC-LDPC codes are separately used at the source and relay. At the relay, the information bits are selected from the decoded source information bits, and the selected bits are further encoded. By combining the two LDPC codewords generated at the source and relay, the destination constructs a channel code corresponding to each information selection.
- For making the destination construct the best code, an optimal information bit selection algorithm by exhaustive search is proposed at the relay to appropriately select partial source information bits. In the exhaustive-based search algorithm, the best pattern is chosen from all selection patterns, during which we consider all source information bit sequences.
- Since all the source information bit sequences and all the selection patterns are considered, the complexity of the optimal algorithm is relatively high when the QC-LDPC codes have large block length. Based on this, another partial-based search information bit selection algorithm is proposed with partial source information bit sequences and partial selection patterns being taken into account.
- At the destination, the joint iterative decoding algorithm based on the three-layer Tanner graph is proposed to effectively recover the source information by the use of the equivalent parity-check matrix.

The proposed optimized selection algorithm chooses an optimized one from the selection patterns considered in the relay, and the proposed joint decoding algorithm based on the three-layer Tanner graph performs single-step decoding by using the equivalent parity-check matrix to fully exchange extrinsic information during each iteration, which helps to significantly enhance the entire system’s performance. However, for larger block-length QC-LDPC codes, the complexity of the proposed algorithms will be increased by considering more selection patterns and a larger size-equivalent parity-check matrix.

The organization of this article is shown below. Section 2 performs the description of the proposed D-QC-LDPCC-SM scheme by selection in the relay. Section 3 proposes two optimized information bit selection algorithms. In Section 4, we describe the joint iterative decoding algorithm on the basis of the three-layer Tanner graph. Section 5 analyzes the simulation results in detail. Finally, we conclude this paper.

*Notation*: Bold italic lowercase and capital letters denote the vector and matrix, respectively. $0_{Z\;\times \;Z}$ is the zero matrix of size Z × Z. [x] is the minimum integer no less than *x*, and “mod” represents the modulo operation. ${\left[\cdot \right]}^{T}$ is used for transpose. ℂ denotes the complex domain. $CN\left(\mu ,\;\sigma^{2}\right)$ denotes the complex Gaussian distribution with mean μ and variance $\sigma^{2}$. $C_{K_{1}}^{K_{2}}$ is a binomial coefficient. |***a***|***b***| denotes the series concatenation of ***a*** and ***b***. |.| denotes the number of elements in a set. 

## 2. Distributed QC-LDPC Coded SM Scheme by Selection in the Relay

This section presents the D-QC-LDPCC-SM scheme by selection in the relay. We first introduce the knowledge of 5G LDPC codes. Additionally, we describe the system model of the proposed scheme. 

### 2.1. 5G LDPC Codes

#### 2.1.1. Preliminaries of QC-LDPC Codes

For QC-LDPC codes, the parity-check matrix is represented as:

(1) $$M=\left[\begin{array}{ccccc} A^{e_{1,1}} & A^{e_{1,2}} & A^{e_{1,3}} & \cdots & A^{e_{1,n_{b}}}\\ A^{e_{2,1}} & A^{e_{2,2}} & A^{e_{2,3}} & \cdots & A^{e_{2,n_{b}}}\\ \vdots & \vdots & \vdots & \ddots & \vdots \\ A^{{}^{e_{m_{b},1}}} & A^{e_{m_{b},2}} & A^{e_{m_{b},3}} & \cdots & A^{e_{m_{b},n_{b}}} \end{array}\right].$$

For $1\;\leq \bar{i\;}\;\leq {\;m}_{b}$ and $1\;\leq \bar{j}\leq {\;n}_{b}$, $e_{\bar{i},\bar{j}}\in \left\{-1,\;0,\;Z\;-\;1\right\}$, in which *Z* denotes the lifting size. In Equation (1), matrix ***A*** is the Z × Z circularly-shifted identity matrix and has the following expression:

(2) $$A=\left[\begin{array}{ccccc} 0 & 1 & 0 & \cdots & 0\\ 0 & 0 & 1 & \cdots & 0\\ \vdots & \vdots & \vdots & \vdots & \vdots \\ 0 & 0 & 0 & \cdots & 1\\ 1 & 0 & 0 & \cdots & 0 \end{array}\right].$$

Matrix $A^{e_{\bar{i},\bar{j}}}$ associated with ***A*** and $e_{\bar{i},\bar{j}}$ in Equation (1) is given by:

(3) $$A^{e_{\bar{i},\bar{j}}}=\{\begin{array}{cc} 0_{Z\times Z} & if\;e_{\bar{i},\bar{j}}=-1\\ {\left(A\right)}^{e_{\bar{i},\bar{j}}} & if\;e_{\bar{i},\bar{j}}\neq -1 \end{array}.$$

The non-negative exponent $e_{\bar{i},\bar{j}}$ of ${\left(A\right)}^{e_{\bar{i},\bar{j}}}$ is called the shift value. All exponents $e_{\bar{i},\bar{j}}$ construct the following exponent matrices:

(4) $$E=\left[\begin{array}{ccccc} e_{1,1} & e_{1,2} & e_{1,3} & \cdots & e_{1,n_{b}}\\ e_{2,1} & e_{2,2} & e_{2,3} & \cdots & e_{2,n_{b}}\\ \vdots & \vdots & \vdots & \ddots & \vdots \\ e_{m_{b},1} & e_{m_{b},2} & e_{m_{b},3} & \cdots & e_{m_{b},n_{b}} \end{array}\right].$$

By the above exponent matrix, the base matrix is directly written by:

(5) $$B=\left[\begin{array}{ccccc} b_{1,1} & b_{1,2} & b_{1,3} & \cdots & b_{1,n_{b}}\\ b_{2,1} & b_{2,2} & b_{2,3} & \cdots & b_{2,n_{b}}\\ \vdots & \vdots & \vdots & \ddots & \vdots \\ b_{m_{b},1} & b_{m_{b},2} & b_{m_{b},3} & \cdots & b_{m_{b},n_{b}} \end{array}\right],\;\mathrm{for}\;b_{\bar{i},\bar{j}}=\{\begin{array}{cc} 0 & if\;e_{\bar{i},\bar{j}}=-1\\ 1 & if\;e_{\bar{i},\bar{j}}\neq -1. \end{array}$$

#### 2.1.2. Characteristics and Encoding of 5G LDPC Codes

**Figure 1:**
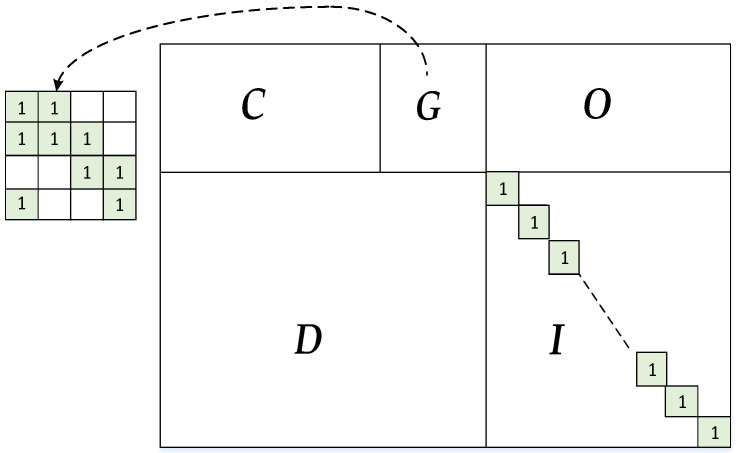
Basic graph of the base matrix.

The 5G LDPC codes adopt the so-called Raptor-like structure, and the base matrix has the sketch depicted in Figure 1. ***C*** and ***G*** construct the core, where ***G*** is a square matrix with the number of elements 1s in the first column being three and the other columns together forming the bidiagonal structure. ***O*** (zero matrix), ***D*** and ***I*** (identity matrix) construct the extension. Moreover, ***C*** corresponds to the information bits, ***G*** corresponds to the parity bits of the core and ***I*** corresponds to the extended parity bits.

Two base matrices, $B_{1}$ and $B_{2}$, with similar structures are supported in 5G LDPC codes. In 5G LDPC codes, 16 exponent matrices are supported, of which 8 exponent matrices correspond to 1 base matrix. The exponent matrices support all lifting sizes, and their relationship is listed in Table 1. For $B_{1}$, the supported information length *K* and the code rate *R* are 308 < K ≤ 8448 and 1/3 ≤ R ≤ 8/9, respectively. For $B_{2}$, 40 ≤ K ≤ 3840 and 1/5 ≤ R ≤ 2/3 are supported, respectively. To adapt variable *K* and *R* (*R* = *K*/*N*) in 5G (*N*, *K*) QC-LDPC codes, i.e., to adapt diverse codeword length *N*, the shortening and puncturing approaches are required. The steps that get *N* transmitted codeword bits are as follows:

**Step 1**: Obtain the base matrix and $k_{z}$ (information circulant columns of the base matrix) for the given *K* and *R*.

(1) For $B_{1}$: $k_{z}=22$.
(2) For $B_{2}$: $k_{z}=10$ if *K* > 640; $k_{z}\;=\;9$ if 560 < K ≤ 640; $k_{z}=8$ if 192 < K ≤ 560. $k_{z}\;=\;6$ if 40 ≤ K ≤ 192;

**Step 2**: Select the minimal *Z* from Table 1 to make $k_{z}Z\;\geq \;K$. By *Z*, we determine matrix $E={\left[e_{\bar{i},\bar{j}}\right]}_{1\leq \bar{i}\leq m_{b},1\leq \bar{j}\leq n_{b}}$ from the eight exponent matrices from Table 1. For $B_{1}$ and $B_{2}$, $\left(m_{b},n_{b}\right)=\left(46,\;68\right)$ and (42, 52), respectively.

**Step 3**: Compute matrix $P\;=\;{\left[p_{\bar{i},\bar{j}}\right]}_{1\leq \bar{i}\leq m_{z},1\leq \bar{j}\leq n_{z}}$ ($n_{z}=[k_{z}/R]+2\;\leq {\;m}_{b}$, $m_{z}{=n}_{z}-k_{z}\leq n_{b}$), where $p_{\bar{i},\bar{j}}=-1$ for $e_{\bar{i},\bar{j}}=-1$, and $p_{\bar{i},\bar{j}}=e_{\bar{i},\bar{j}}$ (mod *Z*) for $e_{\bar{i},\bar{j}}\neq -1$.

**Step 4**: By dispersing each element of ***P*** into a zero matrix or circularly-shifted identity matrix of size Z × Z, the $m_{z}{Z\;\times \;n}_{z}Z$ parity-check matrix ***H*** utilized for the encoding and decoding of (*N*, *K*) LDPC codes will be obtained.

**Figure 2:**
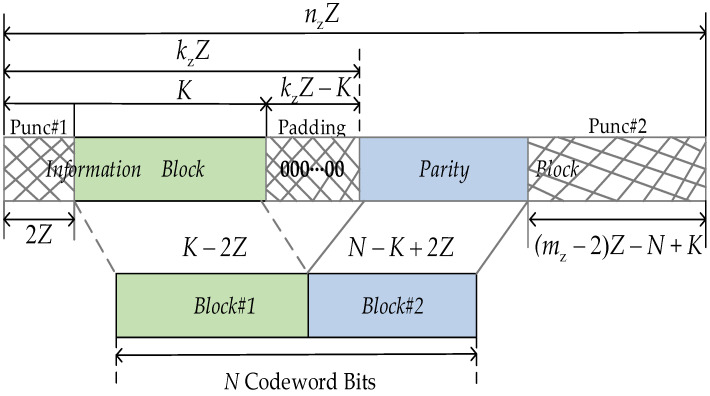
Generation process of *N* transmitted codeword bits.

**Step 5**: Receive the sequence of length $k_{z}Z$ by adding $k_{z}Z\;-\;K$ zero bits at the end of the information sequence of length *K*. Then, we use ***H*** to perform encoding for the obtained sequence of length $k_{z}Z$ to find the codeword sequence ***c*** of length $n_{z}Z$. Finally, the transmitted codeword bits with length *N* are obtained by removing the supplemented zero bits and puncturing partial codeword bits, as exhibited in Figure 2.

**Table 1:** Relationship between the exponent matrices and lifting size sets.

Exponent Matrices	Lifting Size Sets
$E_{1}$	$\left\{Z=a\times 2^{b}|a=2,\;b=0,\;1,\cdots ,\;7\right\}$
$E_{2}$	$\left\{Z=a\times 2^{b}|a=3,\;b=0,\;1,\cdots ,\;7\right\}$
$E_{3}$	$\left\{Z=a\times 2^{b}|a=5,\;b=0,\;1,\cdots ,\;6\right\}$
$E_{4}$	$\left\{Z=a\times 2^{b}|a=7,\;b=0,\;1,\cdots ,\;5\right\}$
$E_{5}$	$\left\{Z=a\times 2^{b}|a=9,\;b=0,\;1,\cdots ,\;5\right\}$
$E_{6}$	$\left\{Z=a\times 2^{b}|a=11,\;b=0,\;1,\cdots ,\;5\right\}$
$E_{7}$	$\left\{Z=a\times 2^{b}|a=13,\;b=0,\;1,\cdots ,\;4\right\}$
$E_{8}$	$\left\{Z=a\times 2^{b}|a=15,\;b=0,\;1,\cdots ,\;4\right\}$

### 2.2. System Model in Cooperative Communications

**Figure 3:**
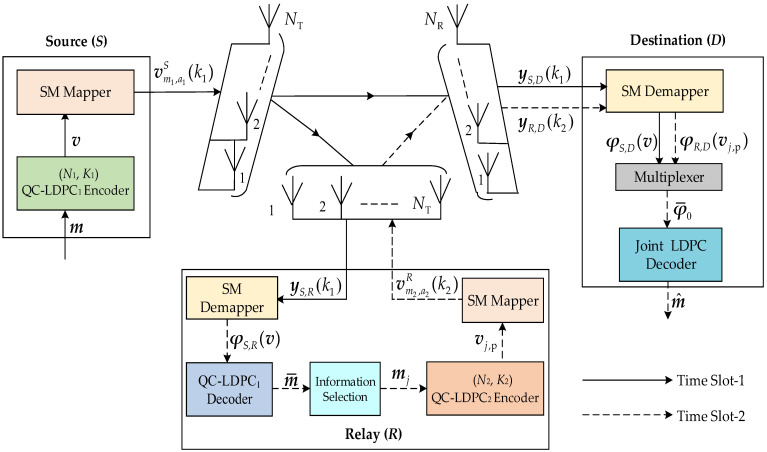
Block diagram of the D-QC-LDPCC-SM scheme.

Figure 3 exhibits the D-QC-LDPCC-SM system model, in which the source (*S*) and relay (*R*) adopt different 5G LDPC codes. The *R* uses the decode-and-forward protocol. Additionally, the *S*, *R* and destination (*D*) deploy *N*_T_, *N*_T_ and *N*_R_ antennas, respectively. Completing a whole system transmission requires two time slots.

**Figure 4:**
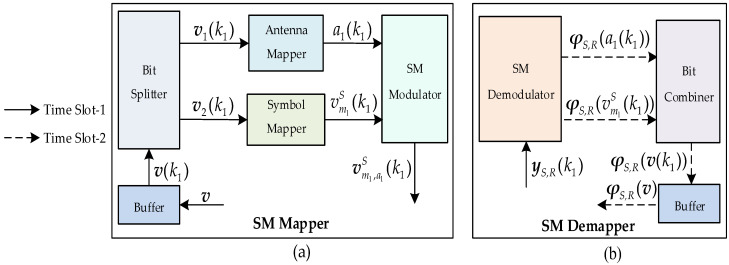
(**a**) SM mapper (**b**) SM demapper.

In time slot-1, the information bit sequence ***m*** with length *K*_1_ at the *S* is given by the 5G (*N*_1_, *K*_1_) QC-LDPC_1_ encoder with $m_{z}^{\left(1\right)}Z^{\left(1\right)}{\;\times \;n}_{z}^{\left(1\right)}Z^{\left(1\right)}$ parity-check matrix ***H***_1_, where $m_{z}^{\left(1\right)}$, $n_{z}^{\left(1\right)}$ and $Z^{\left(1\right)}$ are defined as $m_{z}$, $n_{z}$ and Z, respectively. First, ***m*** is encoded into QC-LDPC codeword sequence $v^{\left(1\right)}$ of length $n_{z}^{\left(1\right)}Z^{\left(1\right)}$. Then, by removing the added zero bits and puncturing the codeword bits, the length of the *N*_1_ transmitted codeword bit sequence ***v*** is generated, as introduced in Section 2.1. Next, ***v*** is sent to the SM mapper to generate a SM vector, and the process is illustrated in Figure 4a. Specifically, the buffer obtains ***v*** and generates multiple length *l* ($l=\log_{2}{(N}_{T}M)$) sequences $v\left(k_{1}\right)$, where *M* is the constellation size, $k_{1}=1,2,\cdots ,(N_{1}\;+\;{\bar{d}}_{1})/l$ with ${\bar{d}}_{1}$ denoting the number of zero bits added at the end of v to make $k_{1}$ an integer. The bit splitter divides $v\left(k_{1}\right)$ into two parts, $v_{1}\left(k_{1}\right)$ and $v_{2}\left(k_{1}\right)$, where $v_{1}\left(k_{1}\right)$ is composed of the first $\log_{2}{(N}_{T})$ bits, but $v_{2}\left(k_{1}\right)$ is composed of the remaining $\log_{2}\left(M\right)$ bits. The antenna mapper finds $v_{1}\left(k_{1}\right)$ and maps it to the transmit antenna index $a_{1}{(k}_{1})\in \left\{1,2,\cdots ,N_{T}\right\}$. The symbol mapper takes $v_{2}\left(k_{1}\right)$ and maps it to the *M*-ary modulated symbol $v_{m_{1}}^{S}{(k}_{1})$, where $m_{1}\in \left\{1,2,\cdots ,\;M\right\}$. Then, the SM modulator gives the modulated symbol $v_{m_{1}}^{S}{(k}_{1})$ to the transmit antenna index $a_{1}{(k}_{1})$, and outputs the transmission vector $v_{m_{1},a_{1}}^{S}\left(k_{1}\right)\in {\mathbb{C}}^{N_{T}\times 1}$:

(6) $$v_{m_{1},a_{1}}^{S}(k_{1})={\left[\cdots ,0,v_{m_{1}}^{S}(k_{1}),0,\cdots \right]}^{T}.$$

The transmission vector $v_{m_{1},a_{1}}^{S}\left(k_{1}\right)$ is separately sent to the *R* and *D* through slow Rayleigh fading channels, $H_{S,R}\in {\mathbb{C}}^{N_{T}\times N_{T}}$ and $H_{S,D}\in {\mathbb{C}}^{N_{R}\times N_{T}}$, to generate the signal vectors $y_{S,R}\left(k_{1}\right)\in {\mathbb{C}}^{N_{T}\times 1}$ and $y_{S,D}\left(k_{1}\right)\in {\mathbb{C}}^{N_{R}\times 1}$:

(7) $$y_{S,R}(k_{1})=H_{S,R}v_{m_{1},a_{1}}^{S}(k_{1})+n_{S,R}(k_{1})=h_{S,R}^{a_{1}(k_{1})}v_{m_{1}}^{S}(k_{1})+n_{S,R}(k_{1})$$

(8) $$y_{S,D}(k_{1})=H_{S,D}v_{m_{1},a_{1}}^{S}(k_{1})+n_{S,D}(k_{1})=h_{S,D}^{a_{1}(k_{1})}v_{m_{1}}^{S}(k_{1})+n_{S,D}(k_{1}),$$

where $h_{S,R}^{a_{1}\left(k_{1}\right)}\in {\mathbb{C}}^{N_{T}\times 1}$ and $h_{S,D}^{a_{1}\left(k_{1}\right)}\in {\mathbb{C}}^{N_{R}\times 1}$ denote the $a_{1}{(k}_{1})\text{-}\mathrm{th}$ column of $H_{S,R}$ and $H_{S,D}$, respectively. $n_{S,R}\left(k_{1}\right)\in {\mathbb{C}}^{N_{T}\times 1}$ and $n_{S,D}\left(k_{1}\right)\in {\mathbb{C}}^{N_{R}\times 1}$ denote the noise vectors. The elements of $H_{S,R}$ and $n_{S,R}\left(k_{1}\right)$ obey the distribution CN(0,1) and $CN\left(0,\sigma^{2}\right)$, respectively. Additionally, $H_{S,D}$ and $n_{S,D}\left(k_{1}\right)$ are defined as $H_{S,R}$ and $n_{S,R}\left(k_{1}\right)$, respectively.

In time slot-2, the SM demapper in *R* is adopted to obtain the log-likelihood ratio (LLR) sequence of ***v***, and the process is illustrated in Figure 4b. Specifically, the SM demodulator using the maximum-likelihood detection approach [21] demodulates the signal $y_{S,R}\left(k_{1}\right)$ to generate the LLR sequences $\varphi_{S,R}\left(a_{1}{(k}_{1})\right)$ and $\varphi_{S,R}\left(v_{m_{1}}^{S}{(k}_{1})\right)$ of $a_{1}{(k}_{1})$ and $v_{m_{1}}^{S}{(k}_{1})$, respectively. By the bit combiner, we receive the LLR sequence $\varphi_{S,R}\left(v\left(k_{1}\right)\right)$. After the buffer, the length *N*_1_ LLR sequence $\varphi_{S,R}\left(v\right)$ of v is generated. The QC-LDPC decoder is used to yield the estimated source information $\bar{m}$. In the *R*, $K_{2}$ ($K_{2}<K_{1}$) information bits $m_{j}$ ($j\in \{1,\;2,\cdots ,{J=C}_{K_{1}}^{K_{2}}\}$) are selected from $\bar{m}$. Note that the proper selection helps the *D* construct an optimized distributed code, and thus designing the optimized information selection algorithms is very important. The details of the optimized algorithms will be introduced in Section 3. Through the 5G (*N*_2_, *K*_2_) QC-LDPC_2_ encoder with $m_{z}^{\left(2\right)}Z^{\left(2\right)}\;\times \;n_{z}^{\left(2\right)}Z^{\left(2\right)}$ parity-check matrix ***H***_2_, $m_{j}$ is encoded into QC-LDPC codeword $v_{j}^{\left(2\right)}=\left[m_{j},v_{j,p}^{\left(2\right)}\right]$ with length $n_{z}^{\left(2\right)}Z^{\left(2\right)}$, where $v_{j,p}^{\left(2\right)}$ has the length $n_{z}^{\left(2\right)}Z^{\left(2\right)}-K_{2}$. We send the length $M_{2}$ ($M_{2}=N_{2}-K_{2}+2Z^{\left(2\right)})$ transmitted codeword bit sequence $v_{j,p}$ of $v_{j,p}^{\left(2\right)}$ to the SM mapper that generates $v_{m_{2},a_{2}}^{R}\left(k_{2}\right)\in {\mathbb{C}}^{N_{T}\times 1}$:

(9) $$v_{m_{2},a_{2}}^{R}(k_{2})={\left[\cdots ,0,v_{m_{2}}^{R}(k_{2}),0,\cdots \right]}^{T},$$

where $k_{2}=1,2,\cdots ,(M_{2}\;+\;{\bar{d}}_{2})/l$ and $v_{m_{2}}^{R}{(k}_{2})$ are transmitted from the $a_{2}{(k}_{2})\text{-}\mathrm{th}$ antenna with $m_{2}\in \left\{1,2,\cdots ,\;M\right\}$ and $a_{2}{(k}_{2})\in \left\{1,\;2,\cdots ,N_{T}\right\}$. Through slow Rayleigh fading channel $H_{R,D}\in {\mathbb{C}}^{N_{R}\times N_{T}}$ (whose definition is similar to $H_{S,R}$ in Equation (7)), vector $v_{m_{2},a_{2}}^{R}\left(k_{2}\right)$ is sent to the *D*, which obtains the signal vector $y_{R,D}\left(k_{2}\right)\in {\mathbb{C}}^{N_{R}\times 1}$:

(10) $$y_{R,D}(k_{2})=H_{R,D}v_{m_{2},a_{2}}^{R}(k_{2})+n_{R,D}(k_{2})=h_{R,D}^{a_{2}(k_{2})}v_{m_{2}}^{R}(k_{2})+n_{R,D}(k_{2}),$$

where $h_{R,D}^{a_{2}\left(k_{2}\right)}\in {\mathbb{C}}^{N_{R}\times 1}$ denotes the $a_{2}{(k}_{2})\text{-}\mathrm{th}$ column of $H_{R,D}$, and the noise vector $n_{R,D}\left(k_{2}\right)\in {\mathbb{C}}^{N_{R}\times 1}$ has a similar definition to $n_{S,R}\left(k_{1}\right)$ in Equation (7).

During the respective time slot, the SM demapper in *D* is used for demodulating the signal vectors $y_{S,D}\left(k_{1}\right)$ and $y_{R,D}\left(k_{2}\right)$ to receive the LLR sequences $\varphi_{S,D}\left(v\right)$ and $\varphi_{R,D}\left(v_{j,p}\right)$ corresponding to v and $v_{j,p}$, respectively. Through the multiplexer, $\varphi_{S,D}\left(v\right)$ and $\varphi_{R,D}\left(v_{j,p}\right)$ are merged into the length $\bar{N}$ ($\bar{N}=N_{1}\;+\;M_{2}$) LLR sequence ${\bar{\varphi }}_{0}=\left[\varphi_{S,D}\left(v\right),\varphi_{R,D}\left(v_{j,p}\right)]=[{\bar{\varphi }}_{0,1},{\bar{\varphi }}_{0,2},\cdots ,{\bar{\varphi }}_{0,\bar{N}}\right]$ corresponding to the transmitted codeword sequence $\left|v\right|v_{j,p}|$. Since $\left|v\right|v_{j,p}|$ are the transmitted bits of distributed LDPC codeword $\left|v^{\left(1\right)}\right|v_{j,p}^{\left(2\right)}|$ with length $\overset{=}{N}=n_{z}^{\left(1\right)}Z^{\left(1\right)}+n_{z}^{\left(2\right)}Z^{\left(2\right)}-K_{2}$, we need to complete the initial LLRs of punctured and shortened bits for ${\bar{\varphi }}_{0}$ to make the joint LDPC decoder find the LLR sequence of length $\overset{=}{N}$. The specific contents of joint LDPC decoding are described in Section 4.

## 3. Optimized Information Bit Selection Algorithms at the Relay

In *R*, we select *K*_2_ bits $m_{j}$ from m with length *K*_1_ as the input of the (*N*_2_, *K*_2_) QC-LDPC_2_ encoder, where *j* is the selection order denoted as follows:

(11) $$j\;\in \;\varepsilon \;=\;\{1,\;2,\;\cdots \;,\;J\;=\;C_{K_{1}}^{K_{2}}\}.$$

Note that each *j* corresponds to a *K*_2_-dimensional vector, i.e.,

(12) $$j↔\psi_{j}=[w_{1}^{\left(j\right)},w_{2}^{\left(j\right)},\cdots ,w_{K_{2}}^{\left(j\right)}],1\leq w_{1}^{\left(j\right)}<w_{2}^{\left(j\right)}<\cdots <w_{K_{2}}^{\left(j\right)}\leq K_{1},$$

where vector $\psi_{j}$ is called the selection pattern, and element $w_{i}^{\left(j\right)}$ represents the position of the selected bit in ***m***. All selection patterns form the following set, mathematically expressed as:

(13) $$\phi =\{\psi_{j}|j\in \varepsilon \}=\{[w_{1}^{\left(j\right)},w_{2}^{\left(j\right)},\cdots ,w_{K_{2}}^{\left(j\right)}],1\leq w_{1}^{\left(j\right)}<w_{2}^{\left(j\right)}<\cdots <w_{K_{2}}^{\left(j\right)}\leq K_{1},j\in \varepsilon \}.$$

For the *j*-th selection, the distributed code at *D* is $C_{D}^{\left(j\right)}(\bar{N},\;K_{1})\;=\;\{|v|v_{j,p}|\}$. Assume that the input sequence of the (*N*_2_, *K*_2_) QC-LDPC_2_ encoder is independent of ***m***, the *D* generates the distributed code $C_{D}(\bar{N},\;K_{1})\;⊇\;C_{D}^{\left(j\right)}(\bar{N},\;K_{1})$.

To obtain an optimized code $C_{D}^{\left(j\right)}(\bar{N},\;K_{1})$ at *D*, two optimized information selection algorithms called the optimal algorithm and the low-complexity algorithm are proposed to appropriately select the partial information from $\bar{m}$. The following optimized selection algorithm description is based on the assumption of correct decoding (i.e., $\bar{m}$ = m) at *R*.

### 3.1. Exhaustive-Based Search Optimal Information Bit Selection Algorithm

In the optimal algorithm based on the exhaustive search, we determine the best pattern resulting in the optimal code with the best codeword weight distribution from all *J* selection patterns, during which all $2^{K_{1}}$ source information bit sequences are considered. The specific steps of the optimal algorithm are listed in Algorithm 1.
**Algorithm 1: Optimal Algorithm.**(1)Determine the set ε and ϕ.(2)Take into account all possible codeword weights $\mathrm{wt}(\left|v\right|v_{j,p}|)\leq \bar{N}$ resulted by $2^{K_{1}}$ source information sequences, where j∈ε.(3)Set the parameters $t=0,\;w=1,\;\phi_{t}=\phi {,\;\varepsilon }_{t}=\varepsilon$.(4)Determine the number $M_{w}^{\left(j\right)}$ of codewords with weight $\mathrm{wt}(\left|v\right|v_{j,p}|)=w$ for each $j\in \varepsilon_{t}\;{\mathrm{and}\;\psi }_{j}\in \phi_{t}$. Find the selection orders *j* causing $\underset{j\;\in \;\varepsilon_{t}}{\min}M_{w}^{\left(j\right)}$ to form a set $\varepsilon_{t+1}\subseteq \varepsilon_{t},$ and then determine the set $\phi_{t+1}\subseteq \phi_{t}$ corresponding to $\varepsilon_{t+1}$.(5)If $w\;<\;\bar{N}$ and $|\phi_{t+1}|\;\neq \;1$, increase the parameters t and *w* by 1, respectively. Then, go to step 4. Otherwise, we terminate the overall search algorithm and obtain the best selection pattern $\psi^{\left(1\right)}=\psi_{j}$.

### 3.2. Partial-Based Search Low-Complexity Information Bit Selection Algorithm

For the case of larger block length code, Algorithm 1 possesses high computational complexity. Based on this, we propose the low-complexity selection algorithm by partial search, different from Algorithm 1, i.e., partial patterns of *J* selection patterns are considered, during which we only consider partial sequences of $2^{K_{1}}$ source information sequences. Algorithm 2 lists the specific design steps.
**Algorithm 2: Low-complexity Algorithm.**(1)Determine the set $\bar{\phi }$ of *Q* (*Q* < *J*) selection patterns, and then get the selection order set $\bar{\varepsilon }$ corresponding to $\bar{\phi }$:
(a)Divide the length *K*_1_ source information bit sequence ***m*** into almost equal two parts, i.e., one part has *L*$\;=[(K_{1}+1)/2]$ bits and the remaining part has $K_{1}-L$ bits. Therefore, there are two cases. For the first case, the number of bits in the first and second parts is *L* and $K_{1}-L$, respectively. For the second case, the first and second parts have $K_{1}-L$ and *L* bits, respectively.(b)We select $K_{2}$ bits from the two parts in each case. For the first case, we randomly choose more bits (i.e., *T* bits) from the first part and fixedly select $K_{2}-T$ bits from the second part, where $([k_{2}+1)/2]\leq T\leq \min\left(K_{2},L\right)$. For the second case, we randomly select *T* bits from the second part, and fixedly select $K_{2}-T$ bits from the first part. Through the selection method, get *Q* selection patterns to construct the set $\bar{\phi }\;=\;\{{\bar{\psi }}_{2},{\bar{\psi }}_{1},\cdots ,{\bar{\psi }}_{Q}\}$.
(c)Get $\bar{\varepsilon }\;=\;\{1,2,\cdots ,Q\}$ corresponding to $\bar{\phi }$.(2)Consider the possible codeword weights $\mathrm{wt}(\left|v\right|v_{j,p}|)\leq \bar{N}$ $\left(j\in \bar{\varepsilon }\right)$ yielded by $K_{b}$ ($K_{b}<2^{K_{1}}$) source information sequences ***m***. The $K_{b}$ information sequences ***m*** are obtained by the following method:
(a)Split the length $K_{1}$ source information sequence *m* into two parts, as shown in substep (a) of step 1.(b)Select $\ell \;(0<\ell \leq \ell_{1}=N_{1}-K_{1}+1)$ positions from the two parts to put non-zero bits, and the remaining $K_{1}-\ell$ positions are put zero bits, where $\ell_{1}$ is the singleton boundary and is related to the minimal codeword weight $d_{\min}\;\mathrm{at}\;\mathrm{the}\;S$, i.e., $d_{\min}\leq N_{1}-K_{1}+1$. The specific selection ways of ℓ positions are similar to substep (b) in step 1. Through the selection method, we get $K_{b}$ sequences ***m*** with weight $0\;<\;\mathrm{wt}(m)\leq N_{1}-K_{1}+1$ (i.e., $0\;<\;\mathrm{wt}(m)\leq d_{\min}$ and $d_{\min}<\;\mathrm{wt}(m)\leq N_{1}-K_{1}+1$). Since the obtained sequences ***m*** easily generate the codewords of low weight at the *S* such that the destination gets low-weight codewords, it is extremely important to consider these sequences ***m***.
(3)Determine the pattern $\psi^{\left(2\right)}={\bar{\psi }}_{j}$ from *Q* selection patterns by referring to steps 3–5 in Algorithm 1.

### 3.3. Complexity Analysis of the Two Optimized Selection Algorithms

This section calculates the encoding complexity of the two optimized algorithms about addition and multiplication.

In Algorithm 1, the complexity of the 5G (*N*_1_, *K*_1_) QC-LDPC_1_ encoder with $m_{z}^{\left(1\right)}Z^{\left(1\right)}{\;\times \;n}_{z}^{\left(1\right)}Z^{\left(1\right)}$ parity-check matrix ***H***_1_ is $m_{z}^{\left(1\right)}Z^{\left(1\right)}\left[{2n}_{z}^{\left(1\right)}Z^{\left(1\right)}-1\right]$ for encoding one information sequence. Then, for $2^{K_{1}}$ information sequences, the overall encoding complexity $J_{1}=2^{K_{1}}m_{z}^{\left(1\right)}Z^{\left(1\right)}\left[{2n}_{z}^{\left(1\right)}Z^{\left(1\right)}-1\right]$ is required at *S*. In *R*, for one selection pattern, the computational complexity required by the 5G (*N*_2_, *K*_2_) QC-LDPC_2_ encoder to complete the encoding of $2^{K_{1}}$ information sequences is $J_{2}=2^{K_{1}}m_{z}^{\left(2\right)}Z^{\left(2\right)}\left[{2n}_{z}^{\left(2\right)}Z^{\left(2\right)}-1\right]$. During determining $\psi^{\left(1\right)}$, assume that the number of the considered selection patterns is separately $B_{1}^{\left(j\right)},\;B_{2}^{\left(j\right)},\;\cdots ,B_{w_{\mathrm{opt}}}^{\left(j\right)}$ for finding $M_{1}^{\left(j\right)},\;M_{2}^{\left(j\right)},\;\cdots ,M_{w_{\mathrm{opt}}}^{\left(j\right)}$. Then, the overall encoding complexity at *R* is $J_{3}=J_{2}\left(B_{1}^{\left(j\right)}+B_{2}^{\left(j\right)}+\cdots +B_{w_{\mathrm{opt}}}^{\left(j\right)}\right)$. Thus, Algorithm 1 has the total complexity denoted as:

(14) $$\begin{array}{ll} \Theta^{\left(1\right)} & =J_{1}+J_{3}\\ & =2^{K_{1}}m_{z}^{\left(1\right)}Z^{\left(1\right)}[2n_{z}^{\left(1\right)}Z^{\left(1\right)}-1]+2^{K_{1}}m_{z}^{\left(2\right)}Z^{\left(2\right)}[2n_{z}^{\left(2\right)}Z^{\left(2\right)}-1](B_{1}^{\left(j\right)}+B_{2}^{\left(j\right)}+\cdots +B_{w_{\mathrm{opt}}}^{\left(j\right)}). \end{array}$$

In Algorithm 2, the total computational complexity is:

(15) $$\Theta^{\left(2\right)}=K_{b}m_{z}^{\left(1\right)}Z^{\left(1\right)}[2n_{z}^{\left(1\right)}Z^{\left(1\right)}-1]+K_{b}m_{z}^{\left(2\right)}Z^{\left(2\right)}[2n_{z}^{\left(2\right)}Z^{\left(2\right)}-1]({\bar{B}}_{1}^{\left(j\right)}+{\bar{B}}_{2}^{\left(j\right)}+\cdots +{\bar{B}}_{w_{\mathrm{low}}}^{\left(j\right)}),$$

where ${\bar{B}}_{1}^{\left(j\right)},{\bar{B}}_{2}^{\left(j\right)},\cdots ,{\bar{B}}_{w_{\mathrm{low}}}^{\left(j\right)}$, separately, are the number of considered selection patterns for finding ${\bar{M}}_{1}^{\left(j\right)},\;{\bar{M}}_{2}^{\left(j\right)},\;\cdots ,{\bar{M}}_{w_{\mathrm{low}}}^{\left(j\right)}$ (defined as $M_{1}^{\left(j\right)},\;M_{2}^{\left(j\right)},\;\cdots ,M_{w_{\mathrm{opt}}}^{\left(j\right)}$) during determining $\psi^{(2)}$. From Equations (14) and (15), the complexity of the two algorithms is obtained.

## 4. Joint Iterative Decoding Algorithm Based on the Three-Layer Tanner Graph

### 4.1. Steps of Joint Iterative Decoding Algorithm

**Figure 5:**
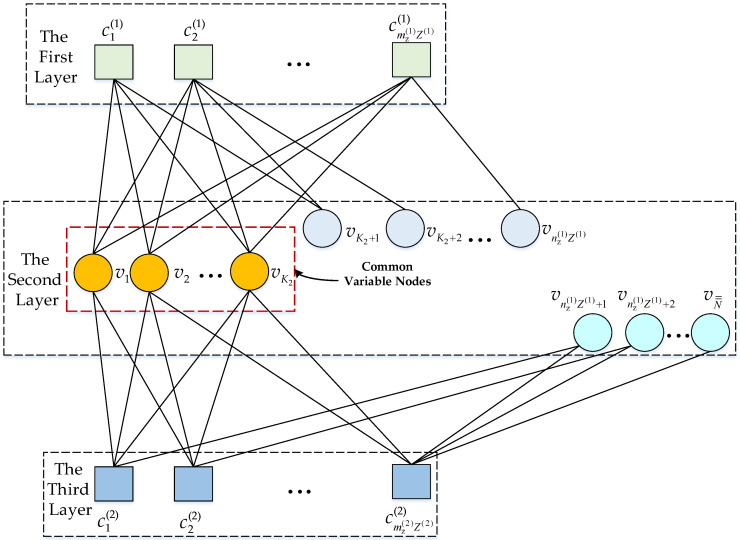
Three-layer Tanner graph of the distributed LDPC code in the *D*.

Joint iterative decoding is another appealing feature of the proposed D-QC-LDPCC-SM scheme. If the first *K*_2_ bits are selected from the *K*_1_ information bits, the equivalent parity-check matrix used for decoding at *D* is expressed as:

(16) $$H_{0}=\left[\begin{array}{cc} {\left(H_{1}\right)}_{m_{z}^{\left(1\right)}Z^{\left(1\right)}\times n_{z}^{\left(1\right)}Z^{\left(1\right)}} & 0_{m_{z}^{\left(1\right)}Z^{\left(1\right)}\times \left(n_{z}^{\left(2\right)}Z^{\left(2\right)}-K_{2}\right)}\\ Q_{m_{z}^{\left(2\right)}Z^{\left(2\right)}\times K_{2}}0_{m_{z}^{\left(2\right)}Z^{\left(2\right)}\times (n_{z}^{\left(1\right)}Z^{\left(1\right)}-K_{2})} & F_{m_{z}^{\left(2\right)}Z^{\left(2\right)}\times \left(n_{z}^{\left(2\right)}Z^{\left(2\right)}-K_{2}\right)} \end{array}\right],$$

where ***H***_1_ is the parity-check matrix of the 5G (*N*_1_, *K*_1_) QC-LDPC_1_ code, and ***H***_2_ = [***Q***, ***F***] is the parity-check matrix of the 5G (*N*_2_, *K*_2_) QC-LDPC_2_ code at *R* with ***Q*** and ***F*** separately having the sizes $m_{z}^{\left(2\right)}Z^{\left(2\right)}\times K_{2}$, $m_{z}^{\left(2\right)}Z^{\left(2\right)}\times \left(n_{z}^{\left(2\right)}Z^{\left(2\right)}-K_{2}\right)$. If the selected $K_{2}$ bits are not in the first $K_{2}$ positions, then matrix ***Q*** is not necessarily in the first $K_{2}$ columns. For the convenience of discussion, we directly use the parity-check matrix $H_{0}$ in Equation (16) to analyze the decoding process, and the corresponding three-layer Tanner graph corresponding to $H_{0}$ is shown in Figure 5. In the three-layer Tanner graph, the check node set $\left\{c_{m_{1}}^{\left(1\right)}{,\;m}_{1}=1,\;2,\cdots ,m_{z}^{\left(1\right)}Z^{\left(1\right)}\right\}$ related to $H_{1}$ forms the first layer. The variable node set $\left\{v_{n_{1}},\;n_{1}=1,2,\cdots ,n_{z}^{\left(1\right)}Z^{\left(1\right)}\right\}$ related to $H_{1}$, and the variable node set $\left\{v_{n_{2}},\;n_{2}=1,\;2,\cdots ,\;K_{2},\;n_{z}^{\left(1\right)}Z^{\left(1\right)}+1,\;n_{z}^{\left(1\right)}Z^{\left(1\right)}+2,\cdots ,\;\overset{=}{N}=n_{z}^{\left(1\right)}Z^{\left(1\right)}+n_{z}^{\left(2\right)}Z^{\left(2\right)}-K_{2}\right\}$ related to $H_{2}$ constitute the second layer of the Tanner graph, where $\left\{v_{\bar{n}},\;\bar{n}=1,\;2,\cdots ,K_{2}\right\}$ is the common variable node set. The check node set $\left\{c_{m_{2}}^{\left(2\right)},\;m_{2}=1,\;2,\cdots ,m_{z}^{\left(2\right)}Z^{\left(2\right)}\right\}$ related to $H_{2}$ constitutes the third layer of the Tanner graph. All check nodes connected with $v_{n}$ ($n=1,\;2,\cdots ,\overset{=}{N}$) constitute the set $C\left(v_{n}\right)$. All variable nodes associated with $c_{m_{i}}^{\left(i\right)}\;(i=1,\;2)$ form the set $V\left(c_{m_{i}}^{\left(i\right)}\right)$. With the help of the three-layer Tanner graph, the decoding steps are as follows:

**Step 1**: Initialize the LLRs of the punctured and shortened bits for ${\bar{\varphi }}_{0}$ to make the decoder find the LLR sequence of length $\overset{=}{N}$, i.e.,

(17) $$\begin{array}{l} \varphi_{0}=[\varphi_{0,1},\varphi_{0,2},\cdots ,\varphi_{0,\overset{=}{N}}]\\ =[\underset{\mathrm{part}\text{-}1}{\underbrace{0,\cdots ,0,}}{\bar{\varphi }}_{0,1},\cdots ,{\bar{\varphi }}_{0,K_{1}-2Z^{\left(1\right)}},\underset{\mathrm{part}\text{-}2}{\underbrace{\infty ,\cdots ,\infty }},{\bar{\varphi }}_{0,K_{1}-2Z^{\left(1\right)}+1},\cdots ,{\bar{\varphi }}_{0,N_{1}},\underset{\mathrm{part}\text{-}3}{\underbrace{0,\cdots ,0,}}\underset{\mathrm{part}\text{-}4}{\underbrace{\infty ,\cdots ,\infty }},{\bar{\varphi }}_{0,N_{1}+1},\cdots ,{\bar{\varphi }}_{0,\bar{N}},\underset{\mathrm{part}\text{-}5}{\underbrace{0,\cdots ,0}}], \end{array}$$

where parts 1, 2 and 3 (related to 5G (*N*_1_, *K*_1_) QC-LDPC_1_ codes) denote the initial LLRs of 2$Z^{\left(1\right)}$ punctured information bits, $k_{z}^{\left(1\right)}Z^{\left(1\right)}-K_{1}$ ($k_{z}^{\left(1\right)}=n_{z}^{\left(1\right)}-m_{z}^{\left(1\right)}$) shortened zero bits and $\left(m_{z}^{\left(1\right)}-2\right)Z^{\left(1\right)}-N_{1}+K_{1}$ punctured parity-check bits, respectively. However, parts 4 and 5 separately denote the initial LLRs of $k_{z}^{\left(2\right)}Z^{\left(2\right)}-K_{2}$ ($k_{z}^{\left(2\right)}=n_{z}^{\left(2\right)}-m_{z}^{\left(2\right)}$) shortened zero bits and $(m_{z}^{\left(2\right)}-2)Z^{\left(2\right)}-N_{2}+K_{2}$ punctured parity-check bits related to 5G (*N*_2_, *K*_2_) QC-LDPC_2_ codes. The extrinsic information ${L(Q}_{m_{i},n}^{\left(i\right)})$ passed by variable node $v_{n}$ to check node $c_{m_{i}}^{\left(i\right)}$ is initialized as $\varphi_{0,n}$.

**Step 2**: The extrinsic information passed by check node $c_{m_{i}}^{\left(i\right)}$ to variable node $v_{n}$ is updated as $L(r_{m_{i},n}^{\left(i\right)})$ by using the iterative decoding algorithm such as belief-propagation (BP) and min-sum (MS) algorithms [22], where $L(r_{m_{i},n}^{\left(i\right)})$ is related to $L(Q_{m_{i},n}^{\left(i\right)})$.

**Step 3**: The extrinsic information $L(Q_{m_{1},n}^{\left(1\right)})$ passed by the variable node $v_{n}$ to the check node $c_{m_{1}}^{\left(1\right)}$ (in the first layer) is updated as follows:

(18) $$L(Q_{m_{1},n}^{\left(1\right)})=\varphi_{0,n}+\sum_{c_{k}^{\left(1\right)}\in C(v_{n})\backslash c_{m_{1}}^{\left(1\right)}}L(r_{k,n}^{\left(1\right)})+\sum_{c_{l}^{\left(2\right)}\in C(v_{n})}L(r_{l,n}^{\left(2\right)}).$$

Similar to $L(Q_{m_{1},n}^{\left(1\right)})$, the extrinsic information $L(Q_{m_{2},n}^{\left(2\right)})$ passed by the variable node $v_{n}$ to the check node $c_{m_{2}}^{\left(2\right)}$ (in the third layer) is updated as follows:

(19) $$L(Q_{m_{2},n}^{\left(2\right)})=\varphi_{0,n}+\sum_{c_{k}^{\left(1\right)}\in C(v_{n})}L(r_{k,n}^{\left(1\right)})+\sum_{c_{l}^{\left(2\right)}\in C(v_{n})\backslash c_{m_{2}}^{\left(2\right)}}L(r_{l,n}^{\left(2\right)}).$$

In (18) and (19), $C\left(v_{n}\right){\backslash c}_{m_{i}}^{\left(i\right)}$ is the set composed of elements other than $c_{m_{i}}^{\left(i\right)}$ in $C\left(v_{n}\right)$.

**Step 4**: Repeat steps 2 and 3. When the maximum number $I_{\max}$ of iterations is reached, the LLR ${L(c}_{n})$ of the *n*-th codeword bit $c_{n}$ and the estimate of $c_{n}$ are exhibited as follows:

(20) $$L(c_{n})=\varphi_{0,n}+\sum_{c_{k}^{\left(1\right)}\in C(v_{n})}L(r_{k,n}^{\left(1\right)})+\sum_{c_{l}^{\left(2\right)}\in C(v_{n})}L(r_{l,n}^{\left(2\right)})$$

(21) $${\hat{c}}_{n}=\{\begin{array}{cc} 0 & L(c_{n})\geq 0\\ 1 & L(c_{n})<0. \end{array}$$

Finally, obtain the estimated information sequence $\hat{m}\;=\;[{\hat{c}}_{1},{\hat{c}}_{2},\cdots ,{\hat{c}}_{K_{1}}]$.

### 4.2. Computational Complexity of Joint Iterative Decoding Algorithm

This subsection considers the computational complexity of the proposed joint iterative decoding algorithm for addition and multiplication. In the parity-check matrix $H_{0}$, let the number of 1′s in the m-th row be $d_{m}$, and the number of 1′s in the n-th column be $\rho_{n}$, where $m\in \beta \;=\;\{1,\;2,\;\cdots ,\;m_{z}^{\left(1\right)}Z^{\left(1\right)}\;+\;m_{z}^{\left(2\right)}Z^{\left(2\right)}\}$ and $n\in \Lambda \;\;=\{1,\;2,\;\cdots ,\;\overset{=}{N}\}$.

First, we consider the complexity of the joint decoding based on the BP algorithm. In step 2, finding the extrinsic information $L(r_{m_{1},n}^{\left(1\right)})$ and $L(r_{m_{2},n}^{\left(2\right)})$ separately needs ${2d}_{k}$ ($k\in \Lambda_{n}^{1}$) and ${2d}_{m_{z}^{\left(1\right)}Z^{\left(1\right)}+l}$ ($l\in \Lambda_{n}^{2}$) elementary operations [22], where $\Lambda_{n}^{1}$ and $\Lambda_{n}^{2}$ represent the index sets of the check nodes in the first and third layers connected to the variable node $v_{n}$, respectively. Note that $|\Lambda_{n}^{1}\left|+|\Lambda_{n}^{2}\right|=\rho_{n}$. In step 3, the number of elementary operations required to find the extrinsic information $L(Q_{m_{i},n}^{\left(i\right)})$ is $\rho_{n}-1$. By combining steps 2 and 3, the complexity required to complete one iteration is computed as:

(22) $$C_{1}^{\mathrm{BP}}=\sum_{n\in \Lambda }\left[\rho_{n}(\rho_{n}-1)+\sum_{k\in \Lambda_{n}^{1}}2d_{k}+\sum_{l\in \Lambda_{n}^{2}}2d_{m_{z}^{\left(1\right)}Z^{\left(1\right)}+l}\right].$$

Since the joint decoding algorithm terminates in the predetermined maximum iteration number $I_{\max}$, the overall computational complexity of the joint BP decoding algorithm is:

(23) $$C^{\mathrm{BP}}=(I_{\max}-1)C_{1}^{\mathrm{BP}}+\sum_{n\in \Lambda }\left[\rho_{n}+\sum_{k\in \Lambda_{n}^{1}}2d_{k}+\sum_{l\in \Lambda_{n}^{2}}2d_{m_{z}^{\left(1\right)}Z^{\left(1\right)}+l}\right].$$

Now, the computational complexity of the joint decoding by the MS algorithm is considered. In step 2, $d_{k}+[\log\left(d_{k}\right)]-2$ ($k\in \Lambda_{n}^{1})$ and $d_{m_{z}^{\left(1\right)}Z^{\left(1\right)}+l}+[\log\left(d_{m_{z}^{\left(1\right)}Z^{\left(1\right)}+l}\right)]-2$ ($l\in \Lambda_{n}^{2})$ elementary operations [22] are required to obtain $L(r_{m_{1},n}^{\left(1\right)})$ and $L(r_{m_{2},n}^{\left(2\right)})$, respectively. Because the MS algorithm is only different from the BP algorithm in step 2, the corresponding complexity required for one iteration can be directly written as:

(24) $$C_{1}^{\mathrm{MS}}=\sum_{n\in \Lambda }\left\{\rho_{n}(\rho_{n}-1)+\sum_{k\in \Lambda_{n}^{1}}\left[d_{k}+\left\lceil \log(d_{k})\right\rceil -2\right]+\sum_{l\in \Lambda_{n}^{2}}\left[d_{m_{z}^{\left(1\right)}Z^{\left(1\right)}+l}+\left\lceil \log\left(d_{m_{z}^{\left(1\right)}Z^{\left(1\right)}+l}\right)\right\rceil -2\right]\right\}.$$

Since the algorithm terminates in the $I_{\max}$-th iteration, the overall computational complexity of the joint MS decoding algorithm is represented as:

(25) $$\begin{array}{l} C^{\mathrm{MS}}\\ =(I_{\max}-1)C_{1}^{\mathrm{MS}}+\sum_{n\in \Lambda }\left\{\rho_{n}+\sum_{k\in \Lambda_{n}^{1}}\left[d_{k}+\left\lceil \log(d_{k})\right\rceil -2\right]+\sum_{l\in \Lambda_{n}^{2}}\left[d_{m_{z}^{\left(1\right)}Z^{\left(1\right)}+l}+\left\lceil \log\left(d_{m_{z}^{\left(1\right)}Z^{\left(1\right)}+l}\right)\right\rceil -2\right]\right\}. \end{array}$$

Based on Equations (23) and (25), we can notice that the joint MS decoding algorithm has a reduced complexity over the joint BP decoding algorithm.

## 5. Simulation Results

The BER performance of the proposed and reference systems over a slow Rayleigh fading channel is discussed. In coded cooperative communications, the signal-to-noise ratio (SNR) of the *S*-*D*, *S*-*R* and *R*-*D* links is represented by $\lambda_{S,D}$, $\lambda_{S,R}$ and $\lambda_{R,D}$, respectively. If $\lambda_{S,R}=\infty$, the *S*-*R* link is ideal, otherwise it is non-ideal. Compared with *S*, *R* is closer to *D*, so let *R* have 1 dB SNR gain, i.e., $\lambda_{R,D}=\lambda_{S,D}+1$. The parameters *N*_T_ = 8 and 16-QAM are used in each simulation. All simulations are reported based on the relationship between $\lambda_{S,D}$ and BER. Table 2 lists the simulation parameters.

### 5.1. Comparisons under Different Information Selection Algorithms

**Figure 6:**
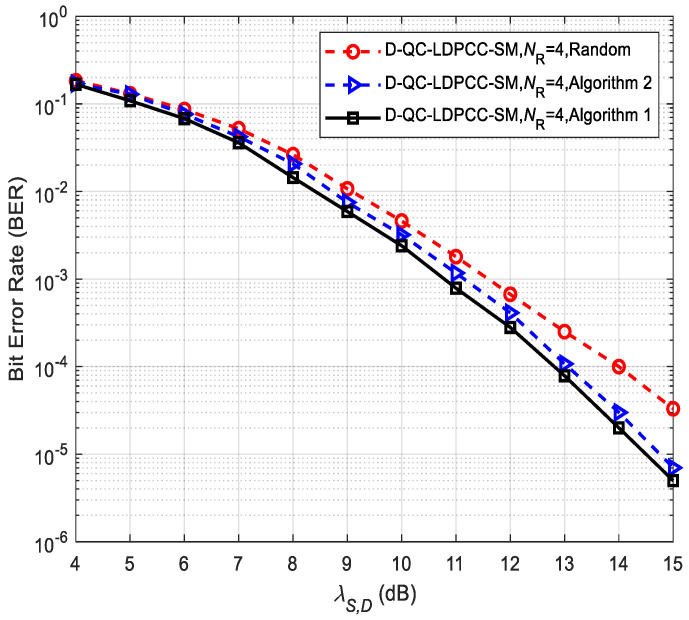
Comparisons of the proposed scheme using the (64, 42) QC-LDPC_1_ and (64, 40) QC-LDPC_2_ codes under different selection algorithms.

To explain the advantages of the proposed optimal and low-complexity information selection algorithms (i.e., Algorithms 1 and 2) over the random selection method, Figure 6 depicts the BER performance of the proposed scheme ($\lambda_{S,R}\;=\;\infty$) with different selection approaches. The corresponding selection patterns are shown in Table 3. The joint BP iterative decoding algorithm is utilized to recover the message. From simulated results, it is noticed that compared with the system utilizing the random selection method, the scheme utilizing the two optimized algorithms provides better performance, which reflects the superiority of our proposed optimized algorithms. For example, at $\mathrm{BER}=3\;\times \;10^{-5}$, Algorithms 1 and 2 outperform the random method by 1 and 1.2 dB, respectively. The reason why the random selection approach provides less performance than the proposed Algorithms 1 and 2 is that it generates the code with a smaller minimum distance of 3 at *D* over the others generating the same minimum distance of 6.

**Figure 7:**
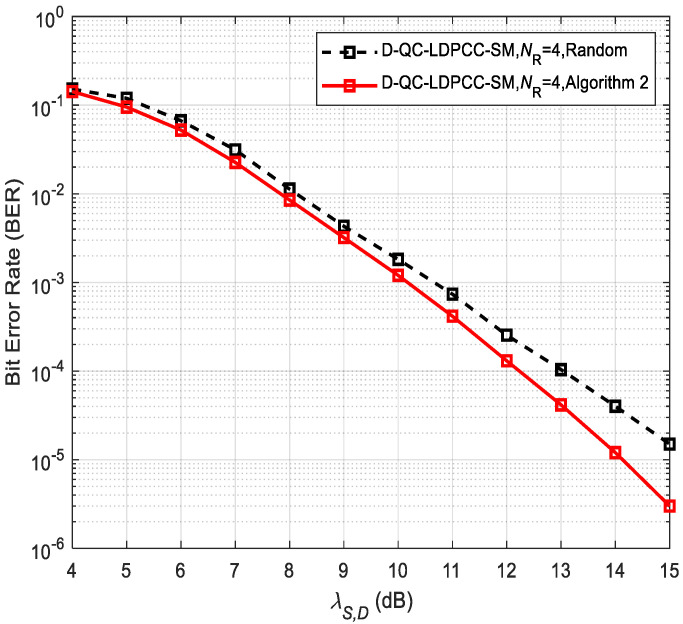
Comparisons of the proposed scheme using the (84, 53) QC-LDPC_1_ and (84, 48) QC-LDPC_2_ codes under different selection approaches.

**Figure 8:**
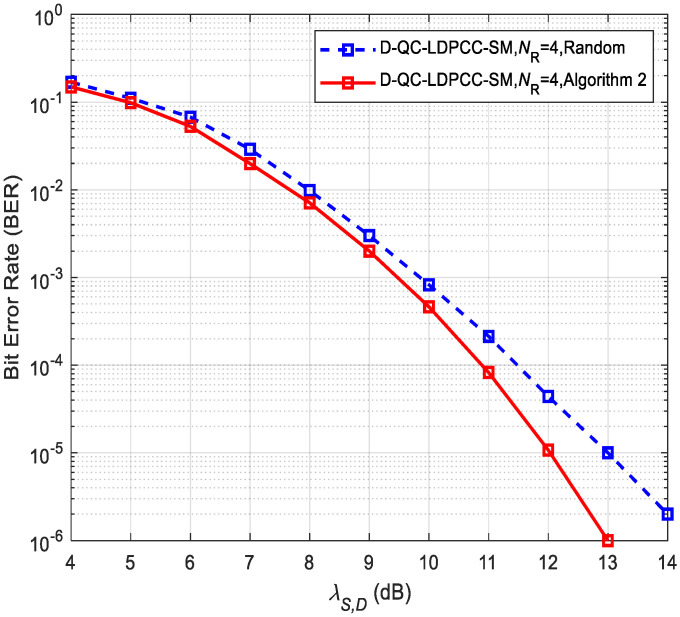
Comparisons of the proposed scheme using the (120, 80) QC-LDPC_1_ and (120, 75) QC-LDPC_2_ codes under different selection approaches.

Moreover, the two optimized algorithms can obtain the approximate performance because they can achieve the code with nearly the same number of codewords with the minimum weight of 6 at *D*. Further, the complexity of Algorithm 2 is greatly reduced over Algorithm 1 by Equations (14) and (15). This reveals the design rationality of low-complexity Algorithm 2. Thus, for the case of the long block length codes, we only concentrate on the impact of Algorithm 2 on the system performance, as exhibited in Figure 7 and Figure 8. Table 3 lists the optimized and random patterns. In the simulations, $\lambda_{S,R}=\infty$ and the joint BP iterative decoding algorithm are assumed. Due to the fact that the random selection method generates a smaller average minimum distance, it again shows the performance degradation of the random approach over Algorithm 2. For example, in Figure 7, the proposed scheme under the random method is about 1.2 dB worse than that under Algorithm 2 at $\mathrm{BER}\approx 10^{-5}$. In Figure 8, the proposed scheme using the random approach has a 1 dB loss compared to the proposed scheme using Algorithm 2 at $\mathrm{BER}=10^{-5}$.

### 5.2. Performance of the Proposed Scheme and Non-Cooperative System

**Figure 9:**
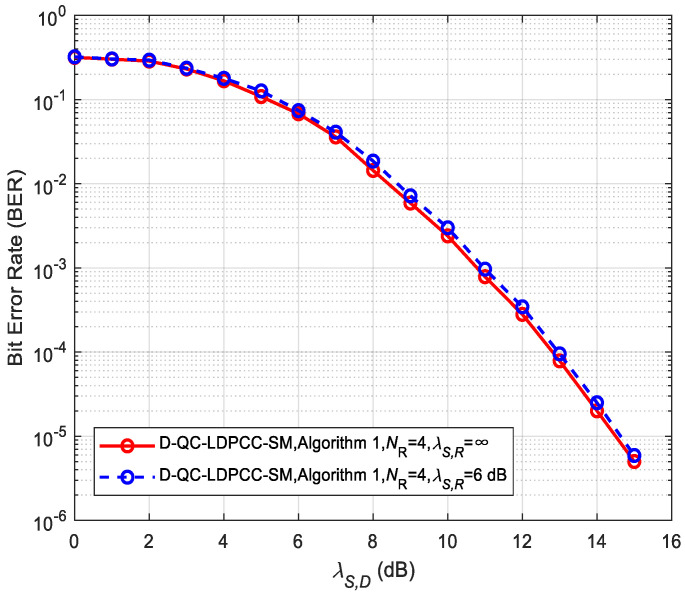
Comparisons of the proposed scheme with Algorithm 1 using (64, 42) QC-LDPC_1_ and (64, 40) QC-LDPC_2_ codes under the ideal and non-ideal *S*-*R* channels.

**Figure 10:**
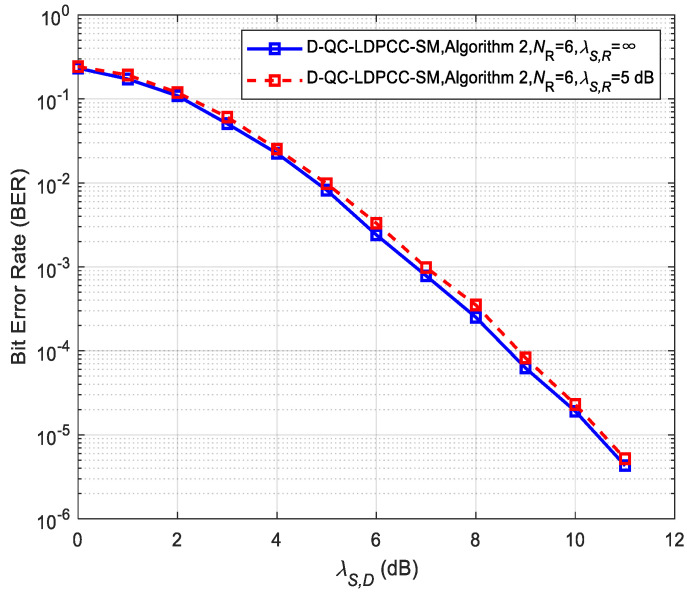
Comparisons of the proposed scheme with Algorithm 2 using (84, 53) QC-LDPC_1_ and (84, 48) QC-LDPC_2_ codes under the ideal and non-ideal channels.

To observe the influence of the practical non-ideal *S*-*R* channel condition ($\lambda_{S,R}\neq \infty$) on the system performance, we carry out the performance comparison for the proposed system in the ideal and non-ideal *S*-*R* channels, as shown in Figure 9 and Figure 10. The joint BP decoding algorithm is utilized. It is noticed that the performance under the non-ideal *S*-*R* channel is very close to that under the ideal *S*-*R* channel. For example, the non-ideal cases in Figure 9 and Figure 10 only have about 0.1 and 0.15 dB losses over the corresponding ideal cases. The results reflect the effectiveness of the proposed system in the realistic wireless channel link. Therefore, studying the proposed D-QC-LDPCC-SM scheme has important theoretical and practical significance.

**Figure 11:**
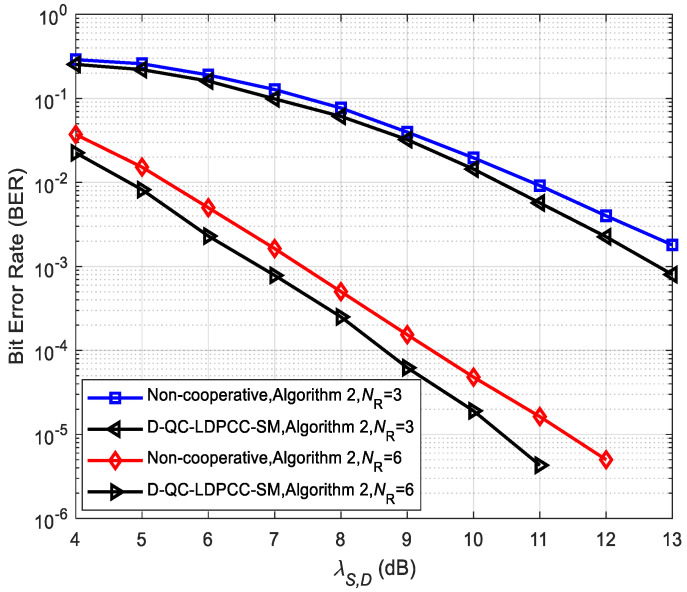
Comparisons of the proposed scheme and non-cooperative system with Algorithm 2 using the (84, 53) QC-LDPC_1_ and (84, 48) QC-LDPC_2_ codes.

Moreover, we perform the performance comparison between the proposed scheme ($\lambda_{S,R}=\infty$) and its corresponding non-cooperative system in Figure 11. At *D*, the joint BP decoding algorithm is used to obtain the estimated source information. We observe that, under the same conditions, the proposed system is significantly better than the non-cooperative system. For example, for the case of *N*_R_ = 6, the cooperative system achieves 0.9 dB over the non-cooperative system at $\mathrm{BER}=10^{-5}$. This is because the larger SNR of the *R*-*D* link over the *S*-*D* link helps to enhance the overall system reliability and further makes the correct estimated probability of the source message be improved.

### 5.3. Comparisons between the Proposed Scheme and the Existing System

**Figure 12:**
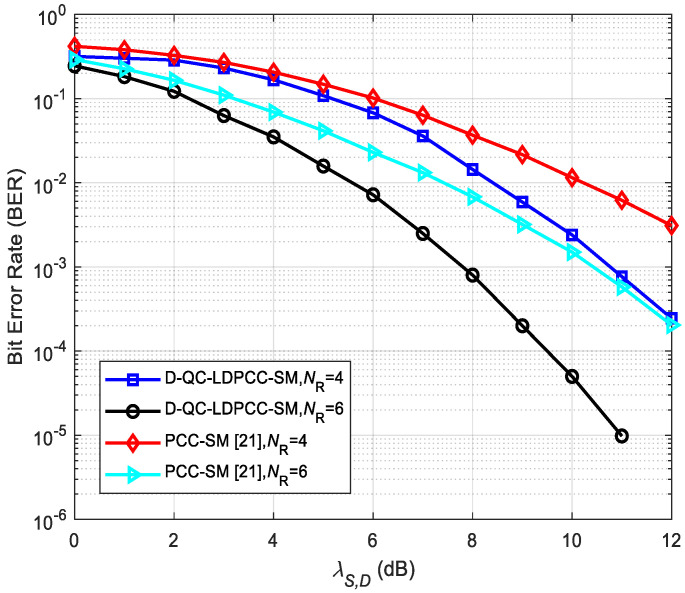
Comparisons between the proposed scheme with Algorithm 1 using the (64, 42) QC-LDPC_1_ and (64, 40) QC-LDPC_2_ codes and the existing system.

In order to better illustrate the effectiveness of the proposed coded cooperative scheme, we compare the proposed scheme with the existing polar-coded cooperative SM (PCC-SM) system [21]. The same conditions, such as $\lambda_{S,R}=\infty$, *N*_T_ = 8 and 16-QAM, are adopted. From Figure 12, we find that the proposed system is superior to the existing PCC-SM system under the same *N*_R_. For example, compared with the PCC-SM system with *N*_R_ = 6, the proposed scheme with *N*_R_ = 6 obtains about 3.1 dB SNR gain at $\mathrm{BER}\approx {2\;\times \;10}^{-4}$. The reasons behind this attractive gain can be attributed to two reasons: (1) The D-QC-LDPCC-SM scheme uses the joint BP decoding algorithm, while the existing PCC-SM system uses successive cancellation decoding without iterations. (2) The proposed scheme utilizes an optimized information selection algorithm, but the existing system selects the subchannel capacities on the basis of the heuristic rather than the optimized method.

### 5.4. Performance of the Proposed Scheme under Various Receive Antenna Numbers and Different Decoding Algorithms

**Figure 13:**
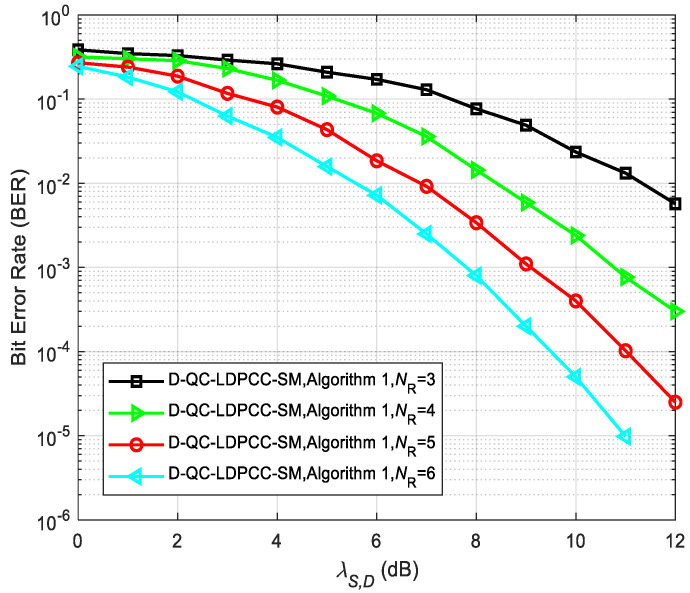
Comparisons between the proposed scheme with Algorithm 1 using the (64, 42) QC-LDPC_1_ and (64, 40) QC-LDPC_2_ codes under various *N*_R_.

**Figure 14:**
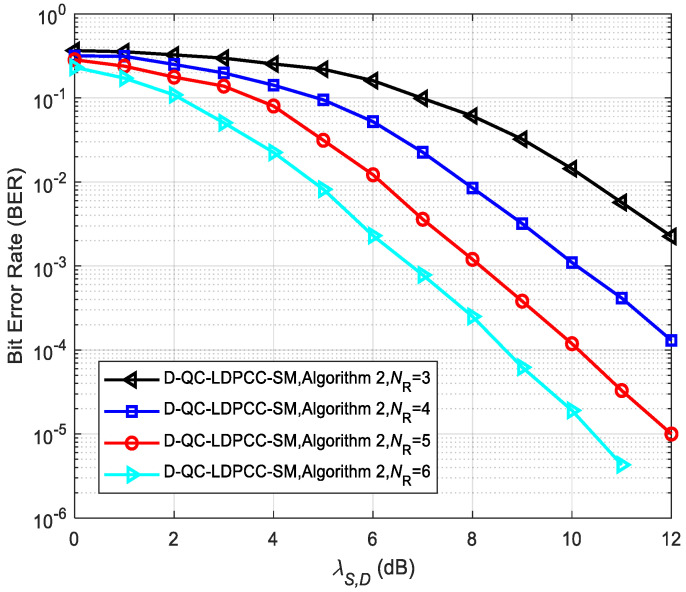
Comparisons between the proposed scheme with Algorithm 2 using the (84, 53) QC-LDPC_1_ and (84, 48) QC-LDPC_2_ codes under various *N*_R_.

Figure 13 and Figure 14 describe the error performance of the proposed system ($\lambda_{S,R}=\infty$) using the joint BP decoding algorithm with different receiving antenna numbers *N*_R_. As depicted in the simulated results, the increase in *N*_R_ greatly improves the BER performance. For example, in Figure 13, the BER performance is $1.3\times 10^{-2}$ with $N_{R}=3$ at SNR = 11 dB. For $N_{R}=4,\;5$ and 6, the BER performance under the same SNR is $7.7\times 10^{-4}$, $1.0\times 10^{-4}$ and $9.8\times 10^{-6}$, respectively. In Figure 14, the BER performance of the proposed system under $N_{R}=3,\;4,\;5$ and 6 is $5.7\times 10^{-3}$, $4.2\times 10^{-4}$, $3.3\times 10^{-5}$ and $4.3\times 10^{-6}$ at SNR = 11 dB. This phenomenon shows that the configuration of more receiving antennas provides more diversity gain for the whole cooperative system, thus enhancing the error performance.

**Figure 15:**
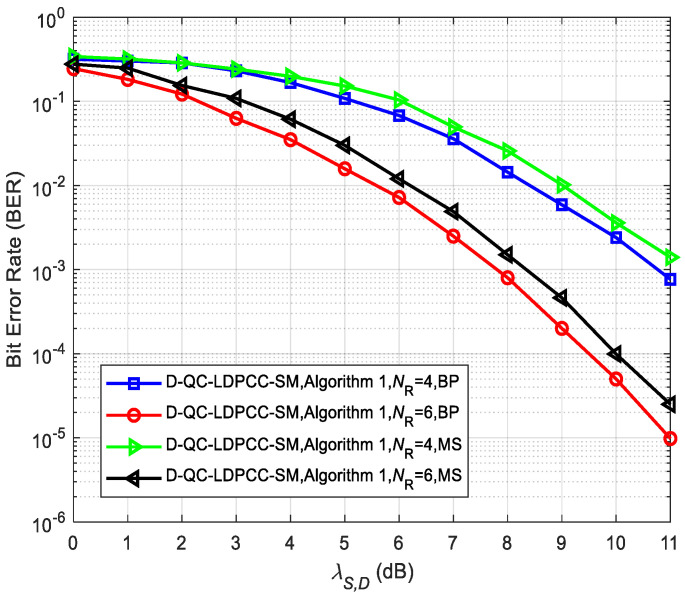
Comparisons of the proposed scheme with Algorithm 1 using the (64, 42) QC-LDPC_1_ and (64, 40) QC-LDPC_2_ codes under different decoding algorithms.

**Figure 16:**
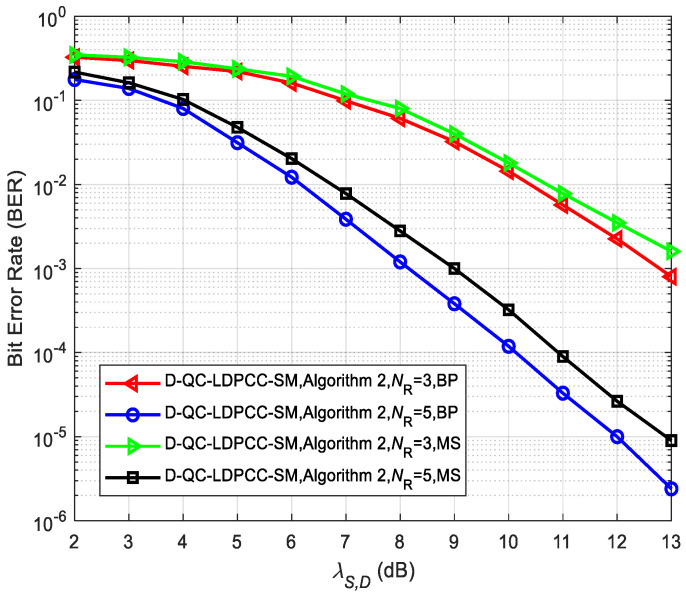
Comparisons of the proposed scheme with Algorithm 2 using the (84, 53) QC-LDPC_1_ and (84, 48) QC-LDPC_2_ codes under different decoding algorithms.

In addition, Figure 15 and Figure 16 compare the system performance ($\lambda_{S,R}=\infty$) under the joint BP and MS decoding algorithms, where the MS decoding algorithm is the simplified BP-based decoding algorithm. It can be found that the performance of the joint MS decoding algorithm is worse than that of the joint BP decoding algorithm. For example, in Figure 15, for the case of *N*_R_ = 6, the MS decoding algorithm lags behind the BP decoding algorithm by about 0.5 dB at $\mathrm{BER}=2.5\times 10^{-5}$. From Figure 16, it is seen that at $\mathrm{BER}=10^{-5}$, the MS decoding algorithm lags behind the BP decoding algorithm by about 0.9 dB for *N*_R_ = 5. The performance loss of the joint MS decoding algorithm is mainly caused by the significant reduction in complexity.

## 6. Conclusions

A novel D-QC-LDPCC-SM scheme is proposed. By adopting the optimized information selection in the relay, the destination generates the optimized code. Compared to the random selection method, the exhaustive-based and partial-based search information bit selection algorithms separately obtain gains of 1 and 1.2 dB due to the obtained larger minimum distance in the destination. In our proposed coded cooperative scheme, the relay-to-destination link has a larger SNR gain than the source-to-destination link, which makes the proposed system exhibit a 0.9 dB gain over the non-cooperative counterpart. Additionally, by using the proper selection and joint iterative decoding algorithm, our proposed scheme outperforms the existing PCC-SM scheme by 3.1 dB.

## Figures and Tables

**Table 2:** Simulation parameters.

Parameters	Specification
Source coding	(64, 42) QC-LDPC_1_ code, (84, 53) QC-LDPC_1_ code, (120, 80) QC-LDPC_1_ code
Relay coding	(64, 40) QC-LDPC_2_ code, (84, 48) QC-LDPC_2_ code, (120, 75) QC-LDPC_2_ code
Channel model	Slow Rayleigh fading channel
SM configuration	$N_{T}=8$$,\;16\text{-}\mathrm{QAM},\;N_{R}=3,\;4$, 5, 6
SM Detection method	Maximum-likelihood detection
Joint iterative decoding algorithm	BP decoding algorithm, MS decoding algorithm
Iteration Number	50

**Table 3:** Selection patterns generated in different approaches.

No.	Selection Approaches	Selection Patterns
1	**Algorithm 1**	[1, 2, 3, 4, 5, 6, 7, 8, 9, 10, 11, 12, 13, 14, 15, 16, 17, 18, 19, 20, 21, 22, 23, 24, 25, 26, 27, 28, 29, 30, 31, 32, 33, 34, 35, 36, 37, 38, 39, 40]
	**Algorithm 2**	[1, 2, 3, 4, 5, 6, 7, 8, 9, 10, 11, 12, 13, 14, 15, 16, 17, 18, 19, 20, 21, 22, 24, 25, 26, 27, 28, 29, 30, 31, 32, 33, 34, 35, 36, 37, 38, 39, 40, 41]
	**Random**	[2, 3, 4, 5, 6, 7, 8, 9, 10, 11, 12, 13, 14, 15, 16, 17, 18, 19, 20, 21, 22, 23, 24, 26, 27, 28, 29, 30, 31, 32, 33, 34, 35, 36, 37, 38, 39, 40, 41, 42]
2	**Algorithm 2**	[1, 2, 3, 4, 5, 6, 7, 8, 9, 10, 12, 13, 14, 15, 16, 17, 18, 19, 20, 21, 22, 23, 25, 26, 27, 28, 29, 30, 31, 32, 33, 34, 35, 36, 37, 38, 39, 40, 42, 43, 44, 45, 46, 47, 48, 49, 50, 51]
	**Random**	[1, 2, 3, 4, 5, 6, 7, 8, 9, 10, 11, 12, 13, 14, 15, 16, 17, 18, 19, 20, 21, 22, 23, 24, 25, 26, 27, 28, 29, 30, 31, 32, 33, 34, 35, 36, 37, 38, 39, 40, 41, 42, 43, 44, 45, 46, 47, 48]
3	**Algorithm 2**	[1, 2, 3, 4, 5, 6, 7, 8, 10, 12, 13, 14, 15, 16, 17, 18, 19, 20, 21, 23, 24, 25, 26, 27, 28, 29, 30, 31, 32, 33, 34, 35, 36, 37, 38, 39, 40, 41, 42, 43, 44, 45, 46, 47, 48, 49, 51, 52, 53, 54, 55, 56, 57, 58, 59, 60, 61, 62, 63, 64, 65, 66, 67, 68, 69, 70, 71, 72, 73, 74, 75, 77, 78, 79, 80]
	**Random**	[1, 2, 3, 4, 5, 6, 7, 8, 10, 12, 13, 14, 15, 16, 17, 18, 19, 20, 21, 23, 24, 25, 26, 27, 28, 29, 30, 31, 32, 33, 34, 35, 36, 37, 38, 39, 40, 41, 42, 43, 44, 45, 46, 47, 48, 49, 50, 51, 52, 53, 54, 55, 56, 57, 58, 59, 60, 61, 62, 63, 64, 65, 66, 67, 68, 69, 70, 71, 72, 73, 74, 75, 76, 77, 78]

## Data Availability

Not applicable.

## References

[1] Hai H., Li C., Peng Y., Hou J., Jiang X. (2021). Space-Time Block Coded Cooperative MIMO Systems. Sensors.

[2] Huang K., Xiao Y., Liu L., Li Y., Song Z., Wang B., Li X. (2022). Integrated Spatial Modulation and STBC-VBLAST Design Toward Efficient MIMO Transmission. Sensors.

[3] Mesleh R.Y., Haas H., Sinanovic S., Ahn C.W., Yun S. (2008). Spatial Modulation. IEEE Trans. Veh. Technol..

[4] Govender R., Pillay N., Xu H. (2014). Soft-Output Space-Time Block Coded Spatial Modulation. IET Commun..

[5] Feng D., Xu H., Zheng J., Bai B. (2018). Nonbinary LDPC-Coded Spatial Modulation. IEEE Trans. Wirel. Commun..

[6] Van Der Meulen E.C. (1971). Three-Terminal Communication Channels. Adv. Appl. Probab..

[7] Zhao C., Yang F., Umar R., Mughal S. (2020). Two-Source Asymmetric Turbo-Coded Cooperative Spatial Modulation Scheme with Code Matched Interleaver. Electronics.

[8] Zhao C., Yang F., Waweru D.K. (2021). Reed-Solomon Coded Cooperative Spatial Modulation Based on Nested Construction for Wireless Communication. Radioengineering.

[9] Sendonaris A., Erkip E., Aazhang B. (2003). User cooperation diversity. Part I. System description. IEEE Trans. Commun..

[10] Sendonaris A., Erkip E., Aazhang B. (2003). User cooperation diversity. Part II. Implementation Aspects and Performance Analysis. IEEE Trans. Commun..

[11] Liang H., Liu A., Liu X., Cheng F. (2021). Construction and Optimization for Adaptive Polar Coded Cooperation. IEEE Wirel. Commun. Lett..

[12] Amirzade F., Sadeghi M.R., Panario D. (2022). QC-LDPC Codes with Large Column Weight and Free of Small Size ETSs. IEEE Commun. Lett..

[13] Hu J., Duman T.M. Low Density Parity Check Codes over Half-duplex Relay Channels. Proceedings of the 2006 IEEE International Symposium on Information Theory.

[14] Fang Y., Liew S.C., Wang T. (2017). Design of Distributed Protograph LDPC Codes for Multi-Relay Coded-Cooperative Networks. IEEE Trans. Wirel. Commun..

[15] Wang H., Chen Q. (2019). LDPC based Network Coded Cooperation Design for Multi-way Relay Networks. IEEE Access.

[16] Berceanu M.G., Voicu C., Halunga S. Uplink Massive MU-MIMO OFDM-Based System with LDPC Coding-Simulation and Performances. Proceedings of the Advanced Topics in Optoelectronics, Microelectronics and Nanotechnologies IX.

[17] Berceanu M.G., Voicu C., Halunga S. The performance of an Uplink Massive MIMO OFDM-Based Multiuser System with LDPC Coding when Using Relays. Proceedings of the 2019 IEEE 25th International Symposium for Design and Technology in Electronic Packaging (SIITME).

[18] Tao X., Chen X., Wang B. (2022). On the Construction of QC-LDPC Codes Based on Integer Sequence with Low Error Floor. IEEE Commun. Lett..

[19] Li H., Bai B., Xu H., Chen C. Construction of Algebraic-Based Variable-Rate QC-LDPC Codes. Proceedings of the 2021 IEEE International Symposium on Information Theory (ISIT).

[20] Amirzade F., Sadeghi M.R., Panario D. Quasi-Cyclic Protograph-Based Raptor-Like LDPC Codes with Girth 6 and Shortest Length. Proceedings of the 2021 IEEE International Symposium on Information Theory (ISIT).

[21] Mughal S., Yang F., Xu H., Umar R. (2018). Coded Cooperative Spatial Modulation Based on Multi-Level Construction of Polar Code. Telecommun. Syst..

[22] Chen J., Dholakia A., Eleftheriou E., Fossorier M.P.C., Hu X.Y. (2005). Reduced-Complexity Decoding of LDPC Codes. IEEE Trans. Commun..

